# Supplementary material for: Kinetics of phagosome maturation is coupled to their intracellular motility
Source: Commun Biol. 2022 Sep 26;5:1014. doi: 10.1038/s42003-022-03988-4 (PMC9512794; doi:10.1038/s42003-022-03988-4)
Supplement: Supplementary file 2 — Description of Additional Supplementary Data [file 42003_2022_3988_MOESM2_ESM.docx]

**Description of Additional Supplementary Files**

**File name:** Supplementary Movie 1

**Description:** Phagosome lysosome fusion without magnetic manipulation 849 monitored by FRET microscopy. Donor emission is shown in magenta and FRET emission is 850 shown in cyan.

**File name:** Supplementary Movie 2

**Description:** . Phagosome lysosome fusion with magnetic manipulation towards 853 nucleus monitored by FRET microscopy. Donor emission is shown in magenta and FRET 854 emission is shown in cyan.
