# Supplementary material for: Kinetics of phagosome maturation is coupled to their intracellular motility
Source: Commun Biol. 2022 Sep 26;5:1014. doi: 10.1038/s42003-022-03988-4 (PMC9512794; doi:10.1038/s42003-022-03988-4)
Supplement: Supplementary file 5 — Reporting Summary-New [file 42003_2022_3988_MOESM5_ESM.pdf]

Corresponding author(s): DBPR, COMMSBIO-22-0966-T

Last updated by author(s): Aug 15, 2022

Reporting Summary

Nature Portfolio wishes to improve the reproducibility of the work that we publish. This form provides structure for consistency and transparency in reporting. For further information on Nature Portfolio policies, see our Editorial Policies and the Editorial Policy Checklist.

Statistics

For all statistical analyses, confirm that the following items are present in the figure legend, table legend, main text, or Methods section.

n/a	Confirmed	
☐	☒	The exact sample size (*n*) for each experimental group/condition, given as a discrete number and unit of measurement
☐	☒	A statement on whether measurements were taken from distinct samples or whether the same sample was measured repeatedly
☐	☒	The statistical test(s) used AND whether they are one- or two-sided<br>*Only common tests should be described solely by name; describe more complex techniques in the Methods section.*
☒	☐	A description of all covariates tested
☒	☐	A description of any assumptions or corrections, such as tests of normality and adjustment for multiple comparisons
☐	☒	A full description of the statistical parameters including central tendency (e.g. means) or other basic estimates (e.g. regression coefficient) AND variation (e.g. standard deviation) or associated estimates of uncertainty (e.g. confidence intervals)
☐	☒	For null hypothesis testing, the test statistic (e.g. *F*, *t*, *r*) with confidence intervals, effect sizes, degrees of freedom and *P* value noted<br>*Give P values as exact values whenever suitable.*
☒	☐	For Bayesian analysis, information on the choice of priors and Markov chain Monte Carlo settings
☒	☐	For hierarchical and complex designs, identification of the appropriate level for tests and full reporting of outcomes
☐	☒	Estimates of effect sizes (e.g. Cohen's *d*, Pearson's *r*), indicating how they were calculated

Our web collection on statistics for biologists contains articles on many of the points above.

Software and code

Policy information about availability of computer code

Data collection	Live cell imaging of phagosome was collected using Epi-Fluorescent Microscopy. Lysotracker Green labeled lysosomes were imaged using Re-scan Confocal Microscopy. The cross-section image of phagosome was collected on a JEOL JEM-1010 Transmission Electron Microscope.
Data analysis	Epi-flourence images in different channels were registered using ImageJ plugin MultistackReg. Particle localization was performed using ImageJ plugin Trackmate. Translational and rotational tracking, wavelet transform analysis, and mean-square displacement analysis were analyzed using customized algorithms in Matlab. Statistical analyses and graph plotting were conducted using OriginPro 2021.

For manuscripts utilizing custom algorithms or software that are central to the research but not yet described in published literature, software must be made available to editors and reviewers. We strongly encourage code deposition in a community repository (e.g. GitHub). See the Nature Portfolio guidelines for submitting code & software for further information.

Data

Policy information about availability of data

All manuscripts must include a data availability statement. This statement should provide the following information, where applicable:

- Accession codes, unique identifiers, or web links for publicly available datasets
- A description of any restrictions on data availability
- For clinical datasets or third party data, please ensure that the statement adheres to our policy

All data collected or analyzed during this study are included in this publication and its supplementary files. Source data for Figure 1. D-I Figure 2. C-F, Figure 3. B-E, Figure 4. C-D, and Figure5. A-E are available as source data file at: https://figshare.com/s/f9e4dfade32cdba8c443

Field-specific reporting

Please select the one below that is the best fit for your research. If you are not sure, read the appropriate sections before making your selection.

☒ Life sciences ☐ Behavioural & social sciences ☐ Ecological, evolutionary & environmental sciences

For a reference copy of the document with all sections, see nature.com/documents/nr-reporting-summary-flat.pdf

Life sciences study design

All studies must disclose on these points even when the disclosure is negative.

Sample size	The sample size of each experiment is provided in the main and supplementary figure captions. No statistical method was used to predetermine the sample size. Number of analyzed single phagosome maturation events were chosen based on similar studies in this field.
Data exclusions	No data were excluded from the analysis
Replication	Each experiments were replicated at least three times with different samples at different days. All replication attempts were successful and all experiments can be reproduced.
Randomization	Randomization was not applicable for this study. However, independent cultures or passages were used for each independent repeat and done on different days. Cells and phagosomes were randomly selected for live-cell imaging.
Blinding	Investigators were not relevant to this study. Analysis of fluorescence intensity and localization of particles were performed by software, independently of human factors.

Reporting for specific materials, systems and methods

We require information from authors about some types of materials, experimental systems and methods used in many studies. Here, indicate whether each material, system or method listed is relevant to your study. If you are not sure if a list item applies to your research, read the appropriate section before selecting a response.

Materials & experimental systems

n/a	Involved in the study	
☐	☒	Antibodies
☐	☒	Eukaryotic cell lines
☒	☐	Palaeontology and archaeology
☒	☐	Animals and other organisms
☒	☐	Human research participants
☒	☐	Clinical data
☒	☐	Dual use research of concern

Methods

n/a	Involved in the study	
☒	☐	ChIP-seq
☒	☐	Flow cytometry
☒	☐	MRI-based neuroimaging

Antibodies

Antibodies used	Alexa Fluor 488 anti-tubulin-α antibody, Alexa Fluor Plus 647 phalloidin were purchased from ThermoFisher (Waltham, MA).
Validation	Antibodies were validated by the manufactures.

Eukaryotic cell lines

Policy information about cell lines

Cell line source(s)	RAW264.7 macrophage were purchased from American Type Culture Collection (ATCC, Rockville, MD). RAW264.7 macrophage stably expressing EGFP-actin was a gift from Prof. Sergio Grinstein at University of Toronto.
Authentication	RAW264.7 macrophage from ATCC was authenticated by ATCC. RAW264.7 macrophage stably expressing EGFP-actin was validated by previous publication (doi: 10.1083/jcb.200412162).
Mycoplasma contamination	All cellines tested negative for mycoplasma prior to experiment.
Commonly misidentified lines (See ICLAC register)	No commonly misidentified cell lines were used in this study.
